# How 40 kilograms of fluid retention can be overlooked: two case reports

**DOI:** 10.1186/1757-1626-2-33

**Published:** 2009-01-08

**Authors:** Hon Shing Ong, Candy Wing-Chiu Sze, Tat Woon Koh, Simon Ward Coppack

**Affiliations:** 1 East London Obesity Service (c/o Dr Simon Coppack), Centre for Diabetes and Metabolic Medicine, Barts and The London School of Medicine, London, E1 2AT, United Kingdom; 2 Department of Cardiology, Barts and The London NHS Trust, London, E1 1BB, United Kingdom

## Abstract

**Introduction:**

With a rising incidence of severe obesity in developed nations, heart failure, a well-recognised co-morbidity, is becoming more common.

**Case presentation:**

We describe two recent patients encountered, a 64 year old and a 42 year old, who are both severely obese and presented with fluid retention of approximately 40 kilograms. Assessment revealed that the explanation of the gross clinical features were relatively subtle cardiac abnormalities. These cases illustrate how fluid retention in severe obesity can differ from that seen in 'traditional' heart failure in terms of clinical assessment and management.

**Conclusion:**

Severe obesity can result in insidious fluid retention, which can be easily overlooked until large volumes of fluid have accumulated. Cardiac abnormalities are usually found in these patients, but may be relatively subtle, leading to current debate in the definition and classification of heart failure. These scenarios are increasingly being encountered in clinical practice. Recognition, assessment and treatment of the 'clinical syndrome of heart failure' in severe obesity is often difficult.

## Introduction

The commonest causes of heart failure are ischaemic heart disease, hypertension, diabetes mellitus, and left ventricular hypertrophy [1]. The management of cardiac failure has evolved to cope with these 'traditional' aetiologies. However, as severe obesity becomes more frequent, so does heart failure, a well-recognised co-morbidity. Unfortunately, awareness of the problem is lagging behind the epidemiology; the British National Institute for Health and Clinical Excellence (NICE) guidelines do not highlight obesity as a potential cause or a contributory factor in heart failure [2]. In addition, the conventional diagnoses of heart failure based on echocardiological findings do not cover the majority of patients with symptoms of cardiac dysfunction. There is currently much debate as to how to define and classify heart failure, with new concepts arising such as 'diastolic heart failure', 'heart failure with normal ejection fraction (HFNEF)', 'acute heart failure syndrome' and 'clinical syndrome of heart failure' [3-5]. Mostly, such diagnostic difficulties occur in elderly patients with multiple co-morbidities, but increasingly severe obesity is another important factor.

We report two recent cases illustrating an increasingly common scenario.

## Case Presentation

### Case 1

A 64 year old Caucasian lady was admitted as an emergency with worsening dyspnoea and oedema. During the three weeks prior to admission she had decreasing exercise tolerance, struggling even to transfer from her bed due to a combination of breathlessness, muscle aches and the sheer weight of her legs. During the preceding nine months, she had three admissions for cellulitis of her swollen legs. Review of the records revealed progressive weight gain of 36 kg over this time.

Her past medical history included type 2 diabetes mellitus, hypertension, and atrial fibrillation, all of which had been controlled on medication for at least 12 months. She also had non-alcoholic steatohepatitis and smoked 5 cigarettes daily. She never had rheumatic fever, heart valvular problems or any cardiovascular events. Her medications were glibenclamide, metformin, co-amilofruse, enalapril, simvastatin, and digoxin.

**Figure 1:**
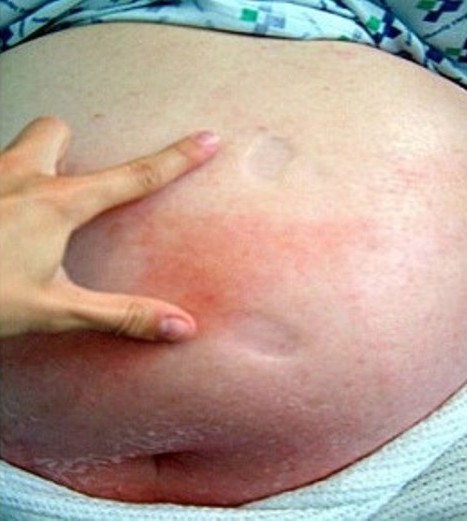
**Photograph showing marked pitting oedema above the umbilicus with areas of cellulitis**.

On examination, she weighed 160.8 kg (equivalent to body mass index (BMI) 60.5 kg/m^2^). There was marked pitting oedema involving both legs, abdomen and chest wall with patches of cellulitis within the oedematous areas (Figure 1). She was haemodynamically stable with a normal venous pressure, impalpable apex beat and normal cardiac auscultation. Chest examination was normal apart from a few bilateral basal crackles. The remaining examination was unremarkable.

Electrocardiogram revealed atrial fibrillation (80 beats/minute) and small QRS complexes. Blood tests were unremarkable. She had evidence of mild proteinuria but no clinical nephrotic syndrome and her renal function was normal. Chest radiography revealed cardiomegaly with bulky hila due to vascular changes. Transthoracic echocardiography showed a normal left ventricular size with an ejection fraction of 65%, bi-atrial enlargement and a dilated right ventricle with good function. Investigations gave no evidence of pulmonary embolus, ascites, sleep disordered breathing or other cause of cardiomyopathy.

She was treated with intravenous furosemide initially and later with metolazone and spironolactone. Her weight reduced by 35.6 kg over 42 days and by 41.8 kg when reviewed in clinic. The patient was not on a strict diet during this period.

### Case 2

A 42 year old Caucasian lady was admitted from an obesity clinic. She had been referred by her general practitioner because of more than 35 kg weight gain over ten months. During this period, she had developed swelling in legs and abdomen but despite furosemide (80 mg daily), the oedema had progressed to render her bedbound.

She had been obese since childhood and depressed recently, but had no other significant medical history. She never had rheumatic fever, diabetes, hypertension, valvular heart problems or any vascular events. Her medications on presentation were furosemide and sertraline. She did not smoke and drank approximately eight units of alcohol a week.

On examination, she weighed 185.1 kg (equivalent BMI 66 kg/m^2^). There was pitting oedema extending up to her abdomen. Her pulse was 100 beats/minute irregularly irregular. Blood pressure was 155/85 mmHg and jugular venous pressure raised to her ear lobes. The apex beat was impalpable. Cardiac auscultation and chest examination were normal. Besides a large paraumbilical hernia, the remaining examination was unremarkable.

Electrocardiogram revealed fast atrial fibrillation and poor R-wave progression in the chest leads. Chest radiograph showed cardiomegaly but normal lung fields (allowing for the quality of the film).

Transthoracic echocardiography showed normal left ventricular size with mild concentric hypertrophy and good systolic function. Right ventricle and both atria were all dilated. A myocardial perfusion scan confirmed ejection fraction of 56% with no evidence of ischaemia. Further tests gave no evidence of pulmonary embolus, sleep disordered breathing or other cause of cardiomyopathy.

During two months treatment with digoxin, warfarin, ramipril and furosemide 250 mg daily, her weight declined by 40.1 kg to 145.0 kg. She was not a restrictive diet during this period. She later underwent successful bariatric surgery and hernia repair.

## Discussion

Over 2% of the adult population in the United Kingdom is morbidly obese and this proportion is increasing. Obesity-related mortality and morbidity are rising. The association between obesity and heart failure is well recognised [6]. Obesity increases many of the established risks for heart failure including hypertension, type 2 diabetes mellitus, and dyslipidaemia. Several mechanisms are recognised for a 'direct' effect of obesity *per se *on heart failure. Indeed, cardiac disease remains the commonest cause of premature death in severe obesity with most deaths occurring below the age of 50 [7]. Both these ladies had severe fluid retention with no other cause apparent apart from relatively mild cardiac abnormalities.

Severe obesity changes the clinical problem of fluid retention. In our cases the only abnormalities identified were cardiac. Heart failure in severe obesity can differ from 'traditional' heart failure in several aspects.

Firstly, the extent of fluid retention is relatively hidden by the sheer physical mass of the patient (as seen in both our patients) and by the gradual and insidious accumulation of fluid. Most severely obese patients have some degree of oedema and display nutrition-related weight changes, so it is difficult to recognise worsening oedema.

Secondly, in severe obesity there can be multiple contributors to the dyspnoea and oedema. Hypertension, diabetes, sleep disordered breathing, 'obesity-related asthma', non-alcoholic fatty liver, focal-segmental glomerulo-sclerosis, low physical fitness levels, thromboembolic disease, lymphoedema, and vitamin deficiencies are all common [8]. Obesity potentiates the sympathetic activation seen in heart failure leading to renin-angiotensin-aldosterone axis activation, resulting in further salt and fluid accumulation [9].

Thirdly, cardiac evaluation of the severely obese patient is technically difficult and interpretation controversial. Severe obesity affects cardiac mass, morphology, and histology [10]. The normal ejection fraction in both our patients is a reflection of the relative insensitivity of this index of cardiac function. In the absence of systolic abnormalities our patients do not satisfy some criteria for heart failure. The mild echocardiological abnormalities in our cases would allow a diagnosis of diastolic dysfunction or HFNEF (heart failure with normal ejection fraction) both more common in severely obese patients. Others would take a more agnostic line and label this 'acute heart failure syndrome' or 'clinical syndrome of heart failure' [4,5]. More sensitive techniques such as myocardial tissue Doppler frequently reveal more subtle abnormalities, even in asymptomatic obese patients [11]. Reliance on ejection fraction as a means of diagnosing heart failure in obese patients only detects a proportion of cases, whilst the majority (like ours) would remain unrecognised. The relatively subtle cardiac abnormalities in our patients may have contributed to the slow and insidious accumulation of fluid. Unfortunately, usual criteria for diagnosing and grading diastolic dysfunction, such as Doppler measurements of transmitral and pulmonary vein flow can have poor diagnostic concordance and discriminatory value [12]. Atrial fibrillation further reduces the sensitivity of echocardiography. Gold standard left heart cathetherisation and evaluation of pressure-volume curves at rest and during exercise are not feasible as an investigative tool for the majority of severely obese patients [13]. Natriuretic peptides (e.g. N-terminal proBNP) are elevated in severe obesity *per se *even without heart failure and may also be elevated in non-cardiac causes of fluid retention [14].

Treatment of heart failure in severe obesity is poorly studied as such patients are routinely excluded from drug trials. Weight reduction reverses haemodynamic abnormalities including left ventricular hypertrophy and diastolic dysfunction in the obese [15]. However, for a patient with 40 kg of fluid overload, one should not await weight reduction from lifestyle changes or anti-obesity drugs. Bariatric surgery is hazardous unless heart failure has been recognised and treated. Appreciation of the large amounts of oedema that can accumulate is important if obese patients are not to be undertreated.

## Conclusion

The profile of heart failure is changing. Our patients illustrate not uncommon scenarios. Severely obese patients can insidiously gain considerable amounts of relatively hidden fluid that can be overlooked until large volumes of fluid have accumulated. Cardiac abnormalities are usually found in such patients, but may be relatively subtle and recognition, quantification, assessment and treatment can be difficult.

## Consent

Written informed consent was obtained from the two patients described for the publication of this case report and any accompanying images. A copy of the written consent is available for review by the Editor-in-Chief of this journal.

## Competing interests

The authors declare that they have no competing interests.

## Authors' contributions

HSO was involved in management of cases, literature search, and writing of article. CS was involved in management of case and writing of article. TK was involved in literature search and writing of article, SC was involved in the idea for article, writing of article and Guarantor. All authors have read and approved the final manuscript.
